# Supplementary material for: Neural mechanisms of modulations of empathy and altruism by beliefs of others’ pain
Source: eLife. 2021 Aug 9;10:e66043. doi: 10.7554/eLife.66043 (PMC8373377; doi:10.7554/eLife.66043)
Supplement: Supplementary file 1. [file elife-66043-supp1.docx]

**Supplementary file1.** Statistical results of the mediation analysis (pain intensity mediated the relationship between decreased BOP and monetary donations) in Experiment 1.

| Variable | *Coeff* | *SE* | *t* | *p* | *LLCI* | *ULCI* |
| --- | --- | --- | --- | --- | --- | --- |
| Regression Model 1 (Total effect of *decreased BOP* on monetary donation) | | | | |  |  |
| Independent: Decreased BOP | -1.742 | 0.366 | -4.793 | < 0.001 | -2.461 | -1.022 |
| Dependent: Monetary donation |  |  |  |  |  |  |
|  |  |  |  |  |  |  |
| Regression Model 2 (Decreased BOP to pain intensity) | | | | |  |  |
| Independent: Decreased BOP | -1.533 | 0.289 | -5.305 | < 0.001 | -2.106 | -0.961 |
| Mediator: Pain intensity |  |  |  |  |  |  |
|  |  |  |  |  |  |  |
| Direct effect of pain intensity on monetary donation | | | | |  |  |
| Mediator: Pain intensity | 0.548 | 0.105 | 5.233 | < 0.001 | 0.340 | 0.755 |
| Dependent: Monetary donation |  |  |  |  |  |  |
|  |  |  |  |  |  |  |
| Remaining direct effect of decreased BOP on monetary donation | | | | |  |  |
| Independent: Decreased BOP | -0.902 | 0.366 | -2.468 | 0.015 | -1.626 | -0.178 |
| Dependent: Monetary donation |  |  |  |  |  |  |
|  |  |  |  |  |  |  |
|  | ***Coeff*** | ***SE*** | ***LLCI95*** | ***ULC195*** |  |  |
| Indirect effect of decreased BOP on monetary donation via pain intensity (bootstrap result) | | | | | | |
| Pain intensity | -0.839 | 0.278 | -1.455 | -0.374 |  |  |

Notes. Confidence intervals for indirect effect are bias-corrected and accelerated;

bootstrap resamples = 5000; N = 60.
