# Supplementary material for: Neural mechanisms of modulations of empathy and altruism by beliefs of others’ pain
Source: eLife. 2021 Aug 9;10:e66043. doi: 10.7554/eLife.66043 (PMC8373377; doi:10.7554/eLife.66043)
Supplement: Supplementary file 2. [file elife-66043-supp2.docx]

**Supplementary file 2.** Statistical results of the mediation analysis (pain intensity mediated the relationship between enhanced BOP and monetary donations) in Experiment 1.

| Variable | *Coeff* | | | *SE* | *t* | *p* | *LLCI* | | *ULCI* |
| --- | --- | --- | --- | --- | --- | --- | --- | --- | --- |
| Regression Model 1 (Total effect of *enhanced BOP* on monetary donation) | | | | | | | |  |  |
| Independent: Enhanced BOP | | 0.913 | | 0.468 | 1.952 | 0.053 | -0.013 | | 1.838 |
| Dependent: Monetary donation | |  | |  |  |  |  | |  |
|  | |  | |  |  |  |  | |  |
| Regression Model 2 (Enhanced BOP to pain intensity) | | | | | | | |  |  |
| Independent: Enhanced BOP | | 0.908 | | 0.299 | 3.035 | 0.003 | 0.316 | | 1.501 |
| Mediator: Pain intensity | |  | |  |  |  |  | |  |
|  | |  | |  |  |  |  | |  |
| Direct effect of pain intensity on monetary donation | | | | | | | |  |  |
| Mediator: Pain intensity | | 0.974 | | 0.113 | 8.630 | < 0.001 | 0.751 | | 1.198 |
| Dependent: Monetary donation | |  | |  |  |  |  | |  |
|  | |  | |  |  |  |  | |  |
| Remaining direct effect of enhanced BOP on monetary donation | | | | | | | |  |  |
| Independent: Enhanced BOP | | 0.028 | | 0.381 | 0.072 | 0.943 | -0.727 | | 0.782 |
| Dependent: Monetary donation | |  | |  |  |  |  | |  |
|  | |  | |  |  |  |  | |  |
|  | | ***Coeff*** | | ***SE*** | ***LLCI95*** | ***ULC195*** |  | |  |
| Indirect effect of enhanced BOP on monetary donation via pain intensity (bootstrap result) | | | | | | | | | |
| Pain intensity | | | 0.885 | 0.320 | 0.314 | 1.563 |  | |  |

Notes. Confidence intervals for indirect effect are bias-corrected and accelerated;

bootstrap resamples = 5000; N = 60.
