# Supplementary material for: Neural mechanisms of modulations of empathy and altruism by beliefs of others’ pain
Source: eLife. 2021 Aug 9;10:e66043. doi: 10.7554/eLife.66043 (PMC8373377; doi:10.7554/eLife.66043)
Supplement: Supplementary file 3. [file elife-66043-supp3.docx]

**Supplementary file 3.** Pain intensity, unpleasantness, and monetary donation (mean ± SD) in Experiment 2.

|  | **Patient** | **0%-effective Patient** | |  | **Patient** | **100%-effective Patient** | |  |
| --- | --- | --- | --- | --- | --- | --- | --- | --- |
|  | **1^st^_Round test** | | **2^nd^_Round test** |  | **1^st^_Round test** | | **2^nd^_Round test** |  |
| **Pain Intensity** | 5.715±1.77 | | 6.935±1.49 |  | 5.656±1.68 | | 5.158±1.57 |  |
| **Unpleasantness** | 4.275±2.00 | | 4.898±2.06 |  | 4.150±2.06 | | 3.656±2.24 |  |
| **Monetary Donation** | 3.435±3.10 | | 4.485±3.33 |  | 3.365±3.04 | | 1.790±2.11 |  |

|  | **Statistic Value** | | **ANOVA** | | | **Simple effect (Effective)** | | | **Simple effect (Phase)** | |
| --- | --- | --- | --- | --- | --- | --- | --- | --- | --- | --- |
|  | **Value** | | **Effectiveness** | **Phase** | **Effectiveness*Phase** | | **0%** | **100%** | **1^st^_Round** | **2^nd^_Round** |
| **Pain Intensity** | | F | 50.946 | 5.106 | 70.205 | | 48.055 | 6.017 | 0.486 | 67.054 |
|  |  | P | <0.001 | 0.028 | <0.001 | | <0.001 | 0.017 | 0.489 | <0.001 |
|  |  | η_p_^2^ | 0.463 | 0.080 | 0.543 | | 0.449 | 0.093 | 0.008 | 0.532 |
|  |  | 90% CI | (0.303, 0.575) | (0.005, 0.202) | (0.392, 0.641) | | (0.287, 0.563) | (0.009, 0.218) | (0, 0.082) | (0.379, 0.631) |
| **Unpleasantness** | | F | 35.615 | 0.277 | 45.494 | | 28.928 | 8.025 | 1.590 | 51.218 |
|  |  | P | <0.001 | 0.600 | <0.001 | | <0.001 | 0.006 | 0.212 | <0.001 |
|  |  | η_p_^2^ | 0.376 | 0.005 | 0.435 | | 0.329 | 0.120 | 0.026 | 0.465 |
|  |  | 90% CI | (0.214, 0.501) | (0, 0.069) | (0.273, 0.551) | | (0.169, 0.459) | (0.020, 0.251) | (0, 0.122) | (0.304, 0.576) |
| **Monetary Donation** | | F | 68.897 | 2.053 | 65.517 | | 23.453 | 34.127 | 1.086 | 70.206 |
|  |  | P | <0.001 | 0.157 | <0.001 | | <0.001 | <0.001 | 0.302 | <0.001 |
|  |  | η_p_^2^ | 0.539 | 0.034 | 0.526 | | 0.284 | 0.366 | 0.018 | 0.543 |
|  |  | 90% CI | (0.387, 0.637) | (0, 0.135) | (0.373, 0.627) | | (0.130, 0.418) | (0.204, 0.492) | (0, 0.106) | (0.392, 0.641) |

Note: Effect size is indexed as the partial eta-squared value. The 90% CIs are reported for partial eta-squared value.
