# Supplementary material for: Neural mechanisms of modulations of empathy and altruism by beliefs of others’ pain
Source: eLife. 2021 Aug 9;10:e66043. doi: 10.7554/eLife.66043 (PMC8373377; doi:10.7554/eLife.66043)
Supplement: Supplementary file 4. [file elife-66043-supp4.docx]

**Supplementary file 4.** Statistical results of the mediation analysis (pain intensity mediated the relationship between decreased BOP and monetary donations) in Experiment 2.

| Variable | *Coeff* | *SE* | *t* | *p* | | | *LLCI* | | *ULCI* |  |
| --- | --- | --- | --- | --- | --- | --- | --- | --- | --- | --- |
| Regression Model 1 (Total effect of *decreased BOP* on monetary donation) | | | | | | |  | |  | |
| Independent: Decreased BOP | -0.054 | 0.011 | -4.721 | < 0.001 | | | -0.077 | | -0.031 | |
| Dependent: Monetary donation |  |  |  |  | | |  | |  | |
|  |  |  |  |  | | |  | |  | |
| Regression Model 2 (Decreased BOP to *pain intensity*) | | | | | | |  | |  | |
| Independent: Decreased BOP | -0.025 | 0.010 | -2.661 | 0.010 | | | -0.443 | | -0.006 | |
| Mediator: Pain intensity |  |  |  |  | | |  | |  | |
|  |  |  |  |  | | |  | |  | |
| Direct effect of pain intensity on monetary donation | | | | | | |  | |  | |
| Mediator: Pain intensity | 0.621 | 0.135 | 4.583 | < 0.001 | | | 0.349 | | 0.892 | |
| Dependent: Monetary donation |  |  |  |  | | |  | |  | |
|  |  |  |  |  | | |  | |  | |
| Remaining direct effect of decreased BOP on monetary donation | | | | | | |  | |  | |
| Independent: Decreased BOP | -0.038 | 0.0104444 | -3.657 | | < 0.001 | | -0.059 | | -0.017 | |
| Dependent: Monetary donation |  |  |  | |  | |  | |  | |
|  |  |  |  | |  | |  | |  | |
|  | ***Coeff*** | ***SE*** | ***LLCI95*** | | ***ULC195*** | |  | |  | |
| Indirect effect of decreased BOP on monetary donation via pain intensity (bootstrap result) | | | | | | | | | | |
| Pain intensity | -0.016 | 0.006 | -0.027 | -0.005 | |  | |  | | |

Notes. Confidence intervals for indirect effect are bias-corrected and accelerated;

bootstrap resamples = 5000; N = 60.
