# Supplementary material for: Neural mechanisms of modulations of empathy and altruism by beliefs of others’ pain
Source: eLife. 2021 Aug 9;10:e66043. doi: 10.7554/eLife.66043 (PMC8373377; doi:10.7554/eLife.66043)
Supplement: Supplementary file 5. [file elife-66043-supp5.docx]

**Supplementary file 5.** Statistical results of the mediation analysis (pain intensity mediated the relationship between enhanced BOP and monetary donations) in Experiment 2.

| Variable | *Coeff* | *SE* | *t* | *p* | *LLCI* | *ULCI* |
| --- | --- | --- | --- | --- | --- | --- |
| Regression Model 1 (Total effect of *enhanced BOP* on monetary donation) | | | | |  |  |
| Independent: Enhanced BOP | -0.011 | 0.011 | -1.017 | 0.314 | -0.033 | 0.011 |
| Dependent: Monetary donation |  |  |  |  |  |  |
|  |  |  |  |  |  |  |
| Regression Model 2 (Enhanced BOP to *pain intensity*) | | | | |  |  |
| Independent: Enhanced BOP | 0.005 | 0.009 | 0.613 | 0.542 | -0.012 | 0.023 |
| Mediator: Pain intensity |  |  |  |  |  |  |
|  |  |  |  |  |  |  |
| Direct effect of pain intensity on monetary donation | | | | |  |  |
| Mediator: Pain intensity | 0.526 | 0.146 | 3.596 | < 0.001 | 0.233 | 0.820 |
| Dependent: Monetary donation |  |  |  |  |  |  |
|  |  |  |  |  |  |  |
| Remaining direct effect of enhanced BOP on monetary donation | | | | |  |  |
| Independent: Enhanced BOP | -0.014 | 0.010 | -1.401 | 0.167 | -0.034 | 0.006 |
| Dependent: Monetary donation |  |  |  |  |  |  |
|  |  |  |  |  |  |  |
|  | ***Coeff*** | ***SE*** | ***LLCI95*** | ***ULC195*** |  |  |
| Indirect effect of enhanced BOP on monetary donation via pain intensity (bootstrap result) | | | | | | |
| Pain intensity | 0.003 | 0.005 | -0.009 | 0.013 |  |  |

Notes. Confidence intervals for indirect effect are bias-corrected and accelerated;

bootstrap resamples = 5000; N = 60.
