# Supplementary material for: Neural mechanisms of modulations of empathy and altruism by beliefs of others’ pain
Source: eLife. 2021 Aug 9;10:e66043. doi: 10.7554/eLife.66043 (PMC8373377; doi:10.7554/eLife.66043)
Supplement: Supplementary file 6. [file elife-66043-supp6.docx]

**Supplementary file 6.** Statistical results of the mediation analysis (unpleasantness mediated the relationship between decreased BOP and monetary donations) in Experiment 2.

| Variable | *Coeff* | *SE* | *t* | *p* | *LLCI* | *ULCI* |
| --- | --- | --- | --- | --- | --- | --- |
| Regression Model 1 (Total effect of *decreased BOP* on monetary donation) | | | | |  |  |
| Independent: Decreased BOP | -0.054 | 0.011 | -4.721 | < 0.001 | -0.077 | -0.031 |
| Dependent: Monetary donation |  |  |  |  |  |  |
|  |  |  |  |  |  |  |
| Regression Model 2 (Decreased BOP to *unpleasantness*) | | | | |  |  |
| Independent: Decreased BOP | -0.008 | 0.009 | -0.874 | 0.386 | -0.025 | 0.010 |
| Mediator: Unpleasantness |  |  |  |  |  |  |
|  |  |  |  |  |  |  |
| Direct effect of unpleasantness on monetary donation | | | | |  |  |
| Mediator: Unpleasantness | 0.195 | 0.173 | 1.125 | 0.265 | -0.152 | 0.542 |
| Dependent: Monetary donation |  |  |  |  |  |  |
|  |  |  |  |  |  |  |
| Remaining direct effect of decreased BOP on monetary donation | | | | |  |  |
| Independent: Decreased BOP | -0.052 | 0.011 | -4.572 | < 0.001 | -0.075 | -0.029 |
| Dependent: Monetary donation |  |  |  |  |  |  |
|  |  |  |  |  |  |  |
|  | ***Coeff*** | ***SE*** | ***LLCI95*** | ***ULC195*** |  |  |
| Indirect effect of decreased BOP on monetary donation via unpleasantness (bootstrap result) | | | | | | |
| Unpleasantness | -0.002 | 0.003 | -0.009 | 0.003 |  |  |

Notes. Confidence intervals for indirect effect are bias-corrected and accelerated;

bootstrap resamples = 5000; N = 60.
