# Supplementary material for: Neural mechanisms of modulations of empathy and altruism by beliefs of others’ pain
Source: eLife. 2021 Aug 9;10:e66043. doi: 10.7554/eLife.66043 (PMC8373377; doi:10.7554/eLife.66043)
Supplement: Supplementary file 7. [file elife-66043-supp7.docx]

**Supplementary file 7.** Statistical results of the mediation analysis (unpleasantness mediated the relationship between enhanced BOP and monetary donations) in Experiment 2.

| Variable | *Coeff* | *SE* | *t* | *p* | *LLCI* | *ULCI* |
| --- | --- | --- | --- | --- | --- | --- |
| Regression Model 1 (Total effect of *enhanced BOP* on monetary donation) | | | | |  |  |
| Independent: Enhanced BOP | -0.011 | 0.011 | -1.017 | 0.314 | -0.033 | 0.011 |
| Dependent: Monetary donation |  |  |  |  |  |  |
|  |  |  |  |  |  |  |
| Regression Model 2 (Enhanced BOP to *unpleasantness*) | | | | |  |  |
| Independent: Enhanced BOP | -0.007 | 0.006 | -1.177 | 0.244 | -0.018 | 0.005 |
| Mediator: Unpleasantness |  |  |  |  |  |  |
|  |  |  |  |  |  |  |
| Direct effect of unpleasantness on monetary donation | | | | |  |  |
| Mediator: Unpleasantness | 0.272 | 0.246 | 1.107 | 0.273 | -0.220 | 0.765 |
| Dependent: Monetary donation |  |  |  |  |  |  |
|  |  |  |  |  |  |  |
| Remaining direct effect of enhanced BOP on monetary donation | | | | |  |  |
| Independent: Enhanced BOP | -0.009 | 0.011 | -0.838 | 0.406 | -0.031 | 0.013 |
| Dependent: Monetary donation |  |  |  |  |  |  |
|  |  |  |  |  |  |  |
|  | ***Coeff*** | ***SE*** | ***LLCI95*** | ***ULC195*** |  |  |
| Indirect effect of enhanced BOP on monetary donation via unpleasantness (bootstrap result) | | | | | | |
| Unpleasantness | -0.002 | 0.003 | -0.007 | 0.004 |  |  |

Notes. Confidence intervals for indirect effect are bias-corrected and accelerated;

bootstrap resamples = 5000; N = 60.
