# Supplementary material for: Neural mechanisms of modulations of empathy and altruism by beliefs of others’ pain
Source: eLife. 2021 Aug 9;10:e66043. doi: 10.7554/eLife.66043 (PMC8373377; doi:10.7554/eLife.66043)
Supplement: Supplementary file 8. [file elife-66043-supp8.docx]

**Supplementary file 8.** Statistical results of reaction times, accuracies, and rating scores (mean ± SD) in Experiment 3.

|  | **Patient** |  | |  | **Actor/Actress** |  | |  |
| --- | --- | --- | --- | --- | --- | --- | --- | --- |
|  | **Neutral** | | **Pain** |  | **Neutral** | | **Pain** |  |
| **Reaction time (ms)** | 662±59 | | 669±63 |  | 663±54 | | 674±58 |  |
| **Accuracy (%)** | 85±9.1 | | 81±11.3 |  | 85±12.9 | | 82±14.9 |  |
| **Pain Intensity** | 1.635±0.88 | | 4.798±0.95 |  | 1.248±0.37 | | 3.671±1.42 |  |
| **BOP Rating** |  | | 2.496±2.51 |  |  | | -2.210±3.25 |  |

|  | **Statistic Value** | | **ANOVA** | | **Simple effect (Identity)** | | | **Simple effect (Expression)** | |
| --- | --- | --- | --- | --- | --- | --- | --- | --- | --- |
|  | **Value** | | **Identity** | **Expression** | **Identity*Expression** | **Patient** | **Actor/Actress** | **Neutral** | **Pain** |
| **RT (ms)** | | F | 0.133 | 6.069 | 0.650 |  |  |  |  |
|  |  | P | 0.718 | 0.020 | 0.427 |  |  |  |  |
|  |  | η_p_^2^ | 0.005 | 0.173 | 0.022 |  |  |  |  |
|  |  | 90% CI | (0, 0.105) | (0.016, 0.360) | (0, 0.162) |  |  |  |  |
| **Accuracy (%)** | | F | 0.030 | 18.092 | 0.379 |  |  |  |  |
|  |  | P | 0.863 | <0.001 | 0.543 |  |  |  |  |
|  |  | η_p_^2^ | 0.001 | 0.384 | 0.013 |  |  |  |  |
|  |  | 90% CI | (0, 0.040) | (0.150, 0.546) | (0, 0.139) |  |  |  |  |
| **Pain Intensity** | | F | 27.410 | 212.242 | 4.905 | 160.493 | 87.707 | 8.183 | 16.029 |
|  |  | P | <0.001 | <0.001 | 0.035 | < 0.001 | < 0.001 | 0.008 | < 0.001 |
|  |  | η_p_^2^ | 0.486 | 0.880 | 0.145 | 0.847 | 0.752 | 0.220 | 0.356 |
|  |  | 90% CI | (0.249, 0.625) | (0.795, 0.913) | (0.006, 0.330) | (0.741, 0.889) | (0.592, 0.821) | (0.036, 0.405) | (0.126, 0.523) |

Note: Effect size is indexed as the partial eta-squared value. The 90% CIs are reported for partial eta-squared value.
