# Supplementary material for: Neural mechanisms of modulations of empathy and altruism by beliefs of others’ pain
Source: eLife. 2021 Aug 9;10:e66043. doi: 10.7554/eLife.66043 (PMC8373377; doi:10.7554/eLife.66043)
Supplement: Supplementary file 9. [file elife-66043-supp9.docx]

**Supplementary file 9.** Statistical results of mean ERP amplitudes (mean ± SD) in Experiment 3.

|  | | **Patient** | | |  | **Actor/Actress** | | | |
| --- | --- | --- | --- | --- | --- | --- | --- | --- | --- |
|  | | **Neutral** | | **Pain** |  | **Neutral** | | **Pain** | |
| **N1 amplitude (μV)** | | -3.774±1.42 | | -3.689±1.55 |  | -3.285±1.67 | | -3.600±1.50 | |
| **P2 amplitude (μV)** | | 2.758±2.95 | | 3.715±3.20 |  | 2.974±2.70 | | 3.110±3.07 | |
| **P310 amplitude (μV)** | | 1.463±3.09 | | 1.686±3.15 |  | 1.902±3.22 | | 1.867±3.18 | |
| **P570 amplitude (μV)** | | 4.952±2.44 | | 4.648±2.38 |  | 4.649±2.40 | | 4.692±2.54 | |
|  | **Statistic Value** | | **ANOVA** | | | | **Simple effect (Identity)** | | |
|  | **Value** | | **Identity** | **Expression** | **Identity*Expression** | | **Patient** | | **Actor/Actress** |

| **N1 (95-115ms)** | **F** | 3.500 | 0.363 | 1.334 |  | |  |
| --- | --- | --- | --- | --- | --- | --- | --- |
|  | **P** | 0.072 | 0.551 | 0.258 |  |  |  |
|  | **η_p_^2^** | 0.108 | 0.012 | 0.044 |  |  |  |
|  | **90% CI** | (0, 0.289) | (0, 0.138) | (0, 0.203) |  |  |  |
| **P2 (175-195ms)** | **F** | 1.188 | 9.917 | 7.490 | 18.059 | 0.334 |  |
|  | **P** | 0.285 | 0.004 | 0.010 | <0.001 | 0.568 |  |
|  | **η_p_^2^** | 0.039 | 0.255 | 0.205 | 0.384 | 0.011 |  |
|  | **90% CI** | (0, 0.195) | (0.055, 0.437) | (0.029, 0.391) | (0.150, 0.546) | (0, 0.135) |  |
| **P310 (280-340ms)** | **F** | 2.633 | 0.386 | 1.239 |  | |  |
|  | **P** | 0.115 | 0.539 | 0.275 |  |  |  |
|  | **η_p_^2^** | 0.083 | 0.013 | 0.041 |  |  |  |
|  | **90% CI** | (0, 0.259) | (0, 0.140) | (0, 0.198) |  |  |  |
| **P570 (500-700ms)** | **F** | 0.679 | 1.663 | 1.880 |  | |  |
|  | **P** | 0.417 | 0.207 | 0.181 |  |  |  |
|  | **η_p_^2^** | 0.023 | 0.054 | 0.061 |  |  |  |
|  | **90% CI** | (0, 0.164) | (0, 0.219) | (0, 0.229) |  |  |  |

Note: Effect size is indexed as the partial eta-squared value. The 90% CIs are reported for partial eta-squared value.
