# Supplementary material for: Neural mechanisms of modulations of empathy and altruism by beliefs of others’ pain
Source: eLife. 2021 Aug 9;10:e66043. doi: 10.7554/eLife.66043 (PMC8373377; doi:10.7554/eLife.66043)
Supplement: Supplementary file 10. [file elife-66043-supp10.docx]

**Supplementary file 10.** Statistical results of reaction times, accuracies, and rating scores (mean ± SD) in Experiment 4.

|  | | | **Tiger Team** | | |  | **Lion Team** | | |
| --- | --- | --- | --- | --- | --- | --- | --- | --- | --- |
|  | | | **Neutral** | | **Pain** |  | **Neutral** | **Pain** | |
| **Reaction time (ms)** | | | 665±65 | | 684±69 |  | 673±63 | 688±63 | |
| **Accuracy (%)** | | | 85±10.8 | | 81±11.1 |  | 83±10.4 | 79±12.0 | |
| **Pain Intensity** | | | 1.135±0.22 | | 5.152±1.02 |  | 1.206±0.25 | 5.108±0.87 | |
|  | **Statistic Value** | | **ANOVA** | | | | |  |  |
|  | **Value** | | **Identity** | **Expression** | **Identity * Expression** | | |  |  |
| **RT**  **(ms)** | **F** | | 0.778 | 54.354 | 0.413 | | |  |  |
|  | **P** | | 0.385 | <0.001 | 0.525 | | |  |  |
|  | **η_p_^2^** | | 0.026 | 0.652 | 0.014 | | |  |  |
|  | **90% CI** | | (0, 0.171) | (0.451, 0.749) | (0, 0.143) | | |  |  |
| **Accuracy (%)** | **F** | | 1.527 | 34.919 | 0.080 | | |  |  |
|  | **P** | | 0.226 | <0.001 | 0.779 | | |  |  |
|  | **η_p_^2^** | | 0.050 | 0.546 | 0.003 | | |  |  |
|  | **90% CI** | | (0, 0.212) | (0.317, 0.671) | (0, 0.090) | | |  |  |

| **Pain Intensity** | **F** | 0.057 | 587.788 | 1.608 |
| --- | --- | --- | --- | --- |
|  | **P** | 0.813 | <0.001 | 0.215 |
|  | **η_p_^2^** | 0.002 | 0.953 | 0.053 |
|  | **90% CI** | (0, 0.073) | (0.918, 0.966) | (0, 0.216) |

Note: Effect size is indexed as the partial eta-squared value. The 90% CIs are reported for partial eta-squared value.
