# Supplementary material for: Neural mechanisms of modulations of empathy and altruism by beliefs of others’ pain
Source: eLife. 2021 Aug 9;10:e66043. doi: 10.7554/eLife.66043 (PMC8373377; doi:10.7554/eLife.66043)
Supplement: Supplementary file 11. [file elife-66043-supp11.docx]

**Supplementary file 11.** Statistical results of mean ERP amplitudes (mean ± SD) in Experiment 4.

|  | | | **Tiger Team** | | |  | **Lion Team** | | |
| --- | --- | --- | --- | --- | --- | --- | --- | --- | --- |
|  | | | **Neutral** | | **Pain** |  | **Neutral** | **Pain** | |
| **N1 amplitude (μV)** | | | -3.284±2.12 | | -3.333±1.97 |  | -3.270±1.95 | -3.814±1.76 | |
| **P2 amplitude (μV)** | | | 3.476±3.32 | | 4.090±3.11 |  | 3.386±3.33 | 4.057±3.03 | |
| **P310 amplitude (μV)** | | | 2.211±2.86 | | 2.650±2.91 |  | 2.114±2.85 | 2.813±2.60 | |
| **P570 amplitude (μV)** | | | 4.378±2.13 | | 4.502±2.02 |  | 4.381±1.88 | 4.410±2.11 | |
|  | **Statistic Value** | | **ANOVA** | | | | |  |  |
|  | **Value** | | **Identity** | **Expression** | **Identity * Expression** | | |  |  |

| **N1 (95-115ms)** | **F** | 1.530 | 2.828 | 2.294 |  |
| --- | --- | --- | --- | --- | --- |
|  | **P** | 0.226 | 0.103 | 0.141 |  |
|  | **η_p_^2^** | 0.050 | 0.089 | 0.073 |  |
|  | **90% CI** | (0, 0.213) | (0, 0.266) | (0, 0.246) |  |
| **P2 (175-195ms)** | **F** | 0.116 | 12.182 | 0.040 |  |
|  | **P** | 0.736 | 0.002 | 0.843 |  |
|  | **η_p_^2^** | 0.004 | 0.296 | 0.001 |  |
|  | **90% CI** | (0, 0.101) | (0.081, 0.473) | (0, 0.053) |  |
| **P310 (280-340ms)** | **F** | 0.075 | 10.742 | 1.201 |  |
|  | **P** | 0.786 | 0.003 | 0.282 |  |
|  | **η_p_^2^** | 0.003 | 0.270 | 0.040 |  |
|  | **90% CI** | (0, 0.088) | (0, 0.451) | (0, 0.196) |  |
| **P570 (500-700ms)** | **F** | 0.087 | 0.503 | 0.382 |  |
|  | **P** | 0.770 | 0.484 | 0.541 |  |
|  | **η_p_^2^** | 0.003 | 0.017 | 0.013 |  |
|  | **90% CI** | (0, 0.092) | (0, 0.151) | (0, 0.140) |  |

Note: Effect size is indexed as the partial eta-squared value. The 90% CIs are reported for partial eta-squared value.
