# Supplementary material for: Neural mechanisms of modulations of empathy and altruism by beliefs of others’ pain
Source: eLife. 2021 Aug 9;10:e66043. doi: 10.7554/eLife.66043 (PMC8373377; doi:10.7554/eLife.66043)
Supplement: Supplementary file 12. [file elife-66043-supp12.docx]

**Supplementary file 12.** Statistical results of reaction times, accuracies, and mean ERP amplitudes (mean ± SD) in Experiment 5.

|  | | | **Patient** | | | |  | **Actor/Actress** | | |
| --- | --- | --- | --- | --- | --- | --- | --- | --- | --- | --- |
|  | | | **Neutral** | | **Pain** | |  | **Neutral** | **Pain** | |
| **RT (ms)** | | | 633±69 | | 637±65 | |  | 652±72 | 660±69 | |
| **Accuracy (%)** | | | 87±11.5 | | 86±10.7 | |  | 87±10.5 | 83±13.9 | |
| **N1 amplitude (μV)** | | | -3.183±1.57 | | -3.055±1.38 | |  | -3.003±1.54 | -3.330±1.44 | |
| **P2 amplitude (μV)** | | | 3.043±3.70 | | 3.978±4.00 | |  | 3.219±3.81 | 3.311±4.13 | |
| **P310 amplitude (μV)** | | | 1.454±3.72 | | 2.244±3.47 | |  | 1.818±3.57 | 2.198±3.25 | |
| **P570 amplitude (μV)** | | | 4.224±2.28 | | 4.569±2.40 | |  | 4.103±2.02 | 4.302±1.93 | |
|  | **Statistic Value** | **rm-ANOVA** | | | | | | | **Simple effect (Identity)** | |
|  | **Value** | **Identity** | | **Condition** | | **Identity * Condition** | | | **Patient** | **Actor/Actress** |
| **RT**  **(ms)** | **F** | 23.988 | | 3.243 | | 0.401 | | |  | |
|  | **P** | <0.001 | | 0.082 | | 0.531 | | |  | |
|  | **η_p_^2^** | 0.453 | | 0.101 | | 0.014 | | |  | |
|  | **90% CI** | (0.215, 0.600) | | (0, 0.280) | | (0, 0.141) | | |  | |
| **Accuracy**  **(%)** | **F** | 1.669 | | 9.449 | | 3.983 | | |  | |
|  | **P** | 0.207 | | 0.005 | | 0.055 | | |  | |
|  | **η_p_^2^** | 0.054 | | 0.246 | | 0.121 | | |  | |
|  | **90% CI** | (0, 0.219) | | (0.050, 0.429) | | (0, 0.304) | | |  | |
| **N1**  **(95-115ms)** | **F** | 0.127 | | 0.541 | | 3.834 | | |  | |
|  | **P** | 0.724 | | 0.468 | | 0.060 | | |  | |
|  | **η_p_^2^** | 0.004 | | 0.018 | | 0.117 | | |  | |
|  | **90% CI** | (0, 0.103) | | (0, 0.154) | | (0, 0.300) | | |  | |
| **P2 (175-195ms)** | **F** | 4.614 | | 11.983 | | 9.494 | | | 17.409 | 0.270 |
|  | **P** | 0.040 | | 0.002 | | 0.004 | | | <0.001 | 0.607 |
|  | **η_p_^2^** | 0.137 | | 0.292 | | 0.247 | | | 0.375 | 0.009 |
|  | **90% CI** | (0.003, 0.322) | | (0.079, 0.470) | | (0.050, 0.429) | | | (0.142, 0.539) | (0, 0.127) |
| **P310 (280-340ms)** | **F** | 1.021 | | 22.976 | | 1.651 | | |  | |
|  | **P** | 0.321 | | <0.001 | | 0.209 | | |  | |
|  | **η_p_^2^** | 0.034 | | 0.442 | | 0.054 | | |  | |
|  | **90% CI** | (0, 0.186) | | (0.204, 0.592) | | (0, 0.218) | | |  | |
| **P570 (500-700ms)** | **F** | 1.039 | | 5.094 | | 0.362 | | |  | |
|  | **P** | 0.317 | | 0.032 | | 0.552 | | |  | |
|  | **η_p_^2^** | 0.035 | | 0.149 | | 0.012 | | |  | |
|  | **90% CI** | (0, 0.187) | | (0.007, 0.335) | | (0, 0.138) | | |  | |

Note: Effect size is indexed as the partial eta-squared value. The 90% CIs are reported for partial eta-squared value.
