# Supplementary material for: Neural mechanisms of modulations of empathy and altruism by beliefs of others’ pain
Source: eLife. 2021 Aug 9;10:e66043. doi: 10.7554/eLife.66043 (PMC8373377; doi:10.7554/eLife.66043)
Supplement: Supplementary file 13. [file elife-66043-supp13.docx]

**Supplementary file 13.** Results of the serial mediation analysis in Experiment 5.

| Variable | *Coeff* | *SE* | *t* | *p* | *LLCI* | *ULCI* |
| --- | --- | --- | --- | --- | --- | --- |
| Regression Model 1 (Total effect of decreased BOP on monetary donation) | | | | |  |  |
| Independent: Decreased BOP | -4.679 | 0.499 | -9.370 | <0.001 | -5.679 | -3.679 |
| Dependent: Monetary donation |  |  |  |  |  |  |
|  |  |  |  |  |  |  |
| Regression Model 2 (Decreased BOP to P2 amplitude) | | | | |  |  |
| Independent: Decreased BOP | -0.843 | -0.286 | -2.955 | 0.005 | -1.415 | -0.272 |
| Mediator: P2 amplitude |  |  |  |  |  |  |
|  |  |  |  |  |  |  |
| Regression Model 3 (Decreased BOP to pain intensity) | | | | | | |
| Independent: Decreased BOP | -2.083 | 0.431 | -4.837 | <0.001 | -2.945 | -1.220 |
| Mediator: Pain intensity |  |  |  |  |  |  |
|  |  |  |  |  |  |  |
| Regression Model 4 (P2 amplitude to pain intensity) | | | | | | |
| Independent: P2 amplitude | 0.544 | 0.185 | 2.947 | 0.005 | 0.174 | 0.914 |
| Dependent: Pain intensity |  |  |  |  |  |  |
|  |  |  |  |  |  |  |
| Direct effect of P2 amplitude on monetary donation | | | | |  |  |
| Mediator: P2 amplitude | -0.260 | 0.224 | -1.163 | 0.250 | -0.703 | 0.188 |
| Dependent: Monetary donation |  |  |  |  |  |  |
|  |  |  |  |  |  |  |
| Direct effect of pain intensity on monetary donation | | | | | | |
| Mediator: Pain intensity | 0.568 | 0.150 | 3.797 | <0.001 | 0.268 | 0.867 |
| Dependent: Monetary donation |  |  |  |  |  |  |
|  |  |  |  |  |  |  |
| Remaining direct effect of decreased BOP on monetary donation | | | | |  |  |
| Independent: Decreased BOP | -3.456 | 0.577 | -5.988 | <0.001 | -4.612 | -2.300 |
| Dependent: Monetary donation |  |  |  |  |  |  |
|  | ***Coeff*** | ***SE*** | ***LLCI95*** | ***ULC195*** |  |  |
| Indirect effect of decreased BOP on monetary donation via P2 amplitude (bootstrap result) | | | | | | |
| P2 amplitude | 0.219 | 0.222 | -0.141 | 0.745 |  |  |
| Indirect effect of decreased BOP on monetary donation via pain intensity (bootstrap result) | | | | | | |
| Pain intensity | -1.182 | 0.393 | -2.048 | -0.510 |  |  |
| Indirect effect of decreased BOP on monetary donation via P2 amplitude × pain intensity (bootstrap result) | | | | | | |
| P2 amplitude × pain intensity | -0.261 | 0.137 | -0.584 | -0.059 |  |  |

Notes. Confidence intervals for indirect effect are bias-corrected and accelerated; bootstrap resamples = 5000; N = 60.
