# Supplementary material for: Neural mechanisms of modulations of empathy and altruism by beliefs of others’ pain
Source: eLife. 2021 Aug 9;10:e66043. doi: 10.7554/eLife.66043 (PMC8373377; doi:10.7554/eLife.66043)
Supplement: Supplementary file 14. [file elife-66043-supp14.docx]

**Supplementary file 14.** Statistical results of reaction times, accuracies and rating scores (mean ± SD) in Experiment 6.

|  |  | **Patient** | |  |  | **Actor/Actress** | |  | |
| --- | --- | --- | --- | --- | --- | --- | --- | --- | --- |
|  | **Neutral** | | **Pain** |  | **Neutral** | | **Pain** | |  |
| **Reaction time (ms)** | 1192±384 | | 1221±389 |  | 1168±370 | | 1147±349 | |  |
| **Accuracy (%)** | 84±11.7 | | 80±12.9 |  | 88±8.1 | | 88±10.8 | |  |
| **Pain Intensity** | 1.456±0.79 | | 4.835±1.22 |  | 1.257±0.38 | | 4.062±1.34 | |  |
| **Unpleasantness** | 1.395±0.76 | | 3.693±1.55 |  | 1.121±0.29 | | 2.984±1.42 | |  |
| **BOP Rating** |  | | 2.776±3.20 |  |  | | -4.890±1.44 | |  |

|  | **Statistic Value** | | **ANOVA** | | **Simple effect (Identity)** | | | **Simple effect (Expression)** | |
| --- | --- | --- | --- | --- | --- | --- | --- | --- | --- |
|  | **Value** | | **Identity** | **Expression** | **Identity*Expression** | **Patient** | **Actor/Actress** | **Neutral** | **Pain** |
| **RT (ms)** | | F | 2.279 | 0.027 | 1.760 |  |  |  |  |
|  |  | P | 0.142 | 0.870 | 0.195 |  |  |  |  |
|  |  | η_p_^2^ | 0.071 | 0.001 | 0.055 |  |  |  |  |
|  |  | 90% CI | (0, 0.239) | (0, 0.035) | (0, 0.218) |  |  |  |  |
| **Accuracy (%)** | | F | 9.920 | 5.695 | 3.908 |  |  |  |  |
|  |  | P | 0.004 | 0.024 | 0.057 |  |  |  |  |
|  |  | η_p_^2^ | 0.248 | 0.160 | 0.115 |  |  |  |  |
|  |  | 90% CI | (0.054, 0.429) | (0.012, 0.343) | (0, 0.295) |  |  |  |  |
| **Pain Intensity** | | F | 10.694 | 261.107 | 5.370 | 227.950 | 146.250 | 2.829 | 9.823 |
|  |  | P | 0.003 | <0.001 | 0.027 | <0.001 | <0.001 | 0.103 | 0.004 |
|  |  | η_p_^2^ | 0.263 | 0.897 | 0.152 | 0.884 | 0.830 | 0.086 | 0.247 |
|  |  | 90% CI | (0.063, 0.442) | (0.825, 0.925) | (0.009, 0.335) | (0.804, 0.916) | (0.717, 0.877) | (0, 0.260) | (0.053, 0.427) |
| Unpleasantness | | F | 8.788 | 86.221 | 3.945 |  |  |  |  |
|  |  | P | 0.006 | <0.001 | 0.056 |  |  |  |  |
|  |  | η_p_^2^ | 0.227 | 0.742 | 0.116 |  |  |  |  |
|  |  | 90% CI | (0.042, 0.409) | (0.582, 0.813) | (0, 0.296) |  |  |  |  |

Note: Effect size is indexed as the partial eta-squared value. The 90% CIs are reported for partial eta-squared value.
