# Supplementary material for: Neural mechanisms of modulations of empathy and altruism by beliefs of others’ pain
Source: eLife. 2021 Aug 9;10:e66043. doi: 10.7554/eLife.66043 (PMC8373377; doi:10.7554/eLife.66043)
Supplement: Supplementary file 15. [file elife-66043-supp15.docx]

**Supplementary file 15.** Number of ERP trials for analyses (mean ± SD) in Experiments 3-5.

|  | **Patient/Tiger Team** | |  | **Actor/Actress/Lion Team** | |
| --- | --- | --- | --- | --- | --- |
|  | **Neutral** | **Pain** |  | **Neutral** | **Pain** |
| **Experiment 3** | 100±11 | 96±13 |  | 100±15 | 98±18 |
| **Experiment 4** | 97±21 | 91±20 |  | 93±21 | 91±21 |
| **Experiment 5** | 106±16 | 105±16 |  | 105±15 | 101±18 |
